# Physical inactivity behaviour of European adolescents between 2013 and 2022

**DOI:** 10.1186/s12889-025-25207-5

**Published:** 2025-11-07

**Authors:** Jorge Lopez-Fernandez, Carlos Serrano, Antonio Hernandez-Martin, María Luisa Martin-Sanchez

**Affiliations:** 1 Department of Sports Sciences, Faculty of Medicine, Health and Sports, Universidad Europea de Madrid Villaviciosa de Odón, Madrid, Spain; 2 Real Madrid Graduate School, Faculty of Medicine, Health and Sports, Universidad Europea de Madrid, Madrid, Spain

**Keywords:** Inactivity, Physical activity, Public health, Surveillance

## Abstract

**Background:**

Adolescents in the European Union (EU) are classified as physically inactive (PIA) when not meeting at least 60 min/day of moderate-to-vigorous physical activity (MVPA) every day of the week. However, the diversity in MVPA engagement among those deemed inactive has received limited attention, despite potential differences in associated health risks.

**Objective:**

To analyse changes in physical inactivity patterns among EU adolescents (2013–2022), by gender.

**Methods:**

Self-reported MVPA and gender data of EU adolescents (≤ 18 years) were obtained from the three latest Eurobarometer surveys on physical activity (2013, 2017, and 2022). Four non-mutually exclusive dichotomous thresholds of physical inactivity were created (meeting vs. not meeting) based on the number of days per week with ≥ 60 min of MVPA: “Totally inactive” (0 days), “Very inactive” (< 3 days), “Quite inactive” (< 5 days), and “Inactive” (< 7 days). Chi-square tests with adjusted residuals were used to compare the PIA levels across years. Z-tests for two proportions were used to assess gender differences within each category and year.

**Results:**

In all years, most adolescents were classified as inactive (> 60%). No differences among years were reported for the totally inactive threshold (~ 15%; *p* > 0.05). A significant decline in the prevalence of very inactive, quite inactive, and inactive adolescents was observed in 2022 (*p* < 0.05). Girls consistently reported higher PIA levels than boys across all categories, except for the totally inactive threshold.

**Conclusion:**

Further effort to reduce the physical inactivity engagement of EU adolescents is needed.

## Introduction

Several policies and strategic initiatives aimed at promoting physical activity (PA) among diverse population groups including adolescents have been led by the World Health Organization (WHO) (e.g., the Global Action Plan on Physical Activity 2018–2030) and the European Commission (e.g., the European Union [EU] Physical Activity Country Factsheets) [1, 2]. These strategies to increase PA among youth in the EU are supported not only by multiple WHO position statements grounded in prior evidence [2, 3] but also by a recent systematic review linking high levels of physical inactivity among adolescents in European countries such as the Czech Republic, Italy, Portugal and Croatia to adverse health outcomes [4]. However, engagement in PA among European adolescents remains low: 81% of those aged 11–17 years fail to meet the recommendation of at least 60 min of moderate-to-vigorous physical activity (MVPA) per day [5], and no improvement in adherence to PA recommendations has been observed in recent years [3, 4]. Moreover, gender differences persist, with lower engagement in PA among girls (around 84.7%) than boys (around 77.6%), reflecting structural inequalities in access to and participation in physical activity according to the gender [5, 6].

Consistent with this picture, EU-wide surveillance over the past decade has documented persistently low compliance with MVPA guidelines among adolescents, with marked age- and gender-related disparities and important between-country variation. For example, harmonised analyses of accelerometer data from 18 European countries (*n* = 47,497) estimated that only about 29% of adolescents met recommended activity levels, with lower prevalence in Southern Europe and consistently higher activity among boys [7]. Recent rounds of the WHO-collaborative Health Behaviour in School-aged Children (HBSC) surveys (2017/18 to 2021/22) likewise report low MVPA, inequities by gender and age, and country-specific patterns [8]. EU Special Eurobarometers also indicate that population‑level participation in sport and PA showed little change between 2014 and 2017, with updated 2022 data confirming continued concerns, including among youth (15–24 years) [9, 10]. Therefore, further efforts to effectively enhance PA among inactive EU youth are still needed.

Beyond structural and policy constraints, one key factor contributing to low PA engagement among children and adolescents, not only in Europe but also globally [6], appears to be the widespread availability of sedentary leisure options, such as smartphones, video games and streaming platforms, which directly compete with or replace regular participation in MVPA [11, 12]. The COVID‑19 pandemic and subsequent lockdowns were another significant factor reducing opportunities for PA among European adolescents. Even if severity and timing of restrictions varied substantially across European countries (e.g. school closures, stay-at-home rules and outdoor allowances) some studies suggest that only 9.3% of adolescents met the WHO guidelines for PA during the pandemic, with Italy, Portugal and Russia among the most affected countries [13], while other studies indicate an average decrease in adherence to PA of approximately 20%, with some countries—such as Spain, Italy and Sweden—reporting reductions in adherence of up to 60% [14, 15]. Moreover, school closures were associated with losses of up to 48 min in total daily physical activity and 12 min in MVPA, particularly among children aged 8–12 years [16] and with an increase in screen time of around 70% among children and adolescents [17] evidencing the importance of physical education in total weekly PA among youth [16]. Although the WHO declared an end to the COVID‑19 Public Health Emergency of International Concern in May 2023 [18], many sedentary habits acquired by European youth during the pandemic may persist [19, 20], or be reflected in data captured by physical inactivity surveillance systems operated by European governments and the European Commission, such as the most recent Eurobarometer on sport and PA released in 2022 [10] so its influence in physical inactivity needs to be considered.

Scientific evidence and WHO PA guidelines indicate that any amount of PA, even below the recommended minimum, is preferable to complete inactivity [3, 21, 22]. Accordingly, while the primary goal is to reduce the high levels of physical inactivity reported among European adolescents [5, 6], achieving even modest increases in MVPA among those below the guideline threshold can represent meaningful progress, as small gains are associated with positive health outcomes [21, 22]. This is particularly relevant in youth, for whom WHO guidelines are comparatively stringent (60 min of MVPA daily) [3] which classify as ‘inactive’ both adolescents who perform little or no MVPA during the week and those who achieve 60 min of MVPA on most days [23]. For these reasons, studying PA beyond the classical binary classification (active vs. inactive) and tracking changes in MVPA engagement over time among ‘inactive’ adolescents may help to detect policy‑relevant, incremental improvements in a context where most EU adolescents fail to meet WHO guidelines [5, 6]. Such evidence can clarify whether current—albeit insufficient—initiatives are benefiting adolescents below the threshold and can inform the design and targeting of future public health policies. However, MVPA profiles among adolescents classified as ‘inactive’ have received little or no attention within the EU, limiting understanding of the problem and, consequently, the capacity to address it effectively.

International initiatives such as the Global Matrix Report Card, which assesses child and adolescent PA across multiple countries may be a useful tool for investigating PA behaviours among inactive adolescents. However, comparability across European nations remains limited because each country measures prevalence and levels of physical activity using different methodologies, with no clear standardisation or systematic distinction between categories of inactivity [24]. This lack of harmonisation makes it difficult to monitor trends at regional level and to assess the impact of common policies in relation to both active and inactive behaviour in the adolescent population. Moreover, while public surveys such as the Special Eurobarometers use statistically representative sampling, the Global Matrix relies on expert assessments and existing data sources, which vary in coverage and do not guarantee national representativeness. Accordingly, although the Special Eurobarometers on Sport and PA among adolescents have sample size limitations and are conducted only every few years [25], their consistent methodology, statistically representative sampling across EU countries, and regular implementation by the European Commission make them a potentially valuable source for monitoring physical activity trends in this population [10]. Therefore, the aim of this study was to analyse changes in the physical inactivity patterns of adolescents in the EU between 2013 and 2022, considering different thresholds of weekly MVPA frequency and comparing results by gender.

## Methods

### Design

This is a descriptive epidemiology study conducted according to the STROBE reporting guidelines [26].

### Data source

Special Eurobarometer surveys on Sports and PA are cross-sectional public opinion polls conducted every few years by the European Commission across all EU countries. They use a multi‑stage, probability sampling design [27]. Microdata, questionnaires and technical documentation are openly available via the European Commission’s Eurobarometer/Open Data portal and the GESIS Eurobarometer Data Service [27]. This study used public‑use microdata from the three latest cross‑sectional Special Eurobarometer surveys on sport and PA: Special Eurobarometer 412 (2013; *n* = 27,919 responses); Special Eurobarometer 472 (2017; *n* = 28,031 responses), and Special Eurobarometer 525 (2022; *n* = 26,569 responses). From every survey the following data was extracted: self-reported MVPA, age and gender.

### Measures

The MVPA levels of European adolescents were determined using the modified version of the International Physical Activity Questionnaire (IPAQ-SF) included in the selected Eurobarometer surveys. The IPAQ-SF has been validated by individuals aged 15–69 [28] and used to report PA levels of European adolescents. Whereas data from the modified IPAQ-SF existing in Special Eurobarometer on Sports and PA have been previously used to determine the physical inactivity (PIA) levels of European adults [27] and adolescents [25].

The three selected Eurobarometer has the duration response truncated to five different fixed possibilities (0 min; 1 to 30 min; 31 to 60 min; 61 to 90 min; 91 to 120 min; more than 120 min). As in previous studies responses “30 min or less” were assumed to mean 15 min; “31 to 60 min” was assumed to mean 45 min; “61 to 90 min” was assumed to mean 75 min; “91 to 120 min” was assumed to mean 105 min; and “more than 120 min” was assumed to mean 120 min [27]. Following the methodology applied in previous research, from the initial 82,519 responses (412 [*n* = 27,919 responses]; 472 [*n* = 28,031 responses]; and 525 [*n* = 26,569 responses]), 714 were excluded because they have not a valid response (i.e., different answer than “don’t know” or “error”) for intensity and duration of at least one intensity (i.e., vigorous PA, moderate PA or walking) [27]. From the remaining 81,805 responses, 2,652 responses were removed because they belonged to the United Kingdom region (the UK and Northern Irland) and no responses from this region was included in the Eurobarometer 525 as the UK was no longer part of the EU. Finally, data from adults ≥ 18 years were excluded (*n* = 77,583) obtaining a final dataset of 1,570 cases.

Four non-mutually exclusive dichotomous thresholds of PIA were created (meeting vs. not meeting) based on the number of days per week with ≥ 60 min of MVPA performed by the EU adolescents: “Totally inactive” (0 days), “Very inactive” (< 3 days), “Quite inactive” (< 5 days), and “Inactive” (< 7 days). According to previous research on adolescents [25] walking intensity was not considered because WHO guidelines for PA only considers moderate and vigorous PA [3].

### Statistical analysis

Descriptive statistics for the four PIA thresholds are presented as proportions (%) with corresponding 95% confidence intervals (95% CI). A chi-square (χ²) test was used to examine differences in PIA patterns among EU adolescents across the three time points (2013, 2017, and 2022) setting *p*-value < 0.05. When a significant association was detected, adjusted standardized residuals were examined to identify the specific differences contributing to the overall result. Significant differences were determined by Z-scores greater than 1.96 or less than − 1.96, with a *p*-value < 0.05. Both analyses were conducted using the Statistical Package for the Social Sciences (SPSS), version 29.0 (IBM Corp., Armonk, NY, USA). Finally, Z-tests for two population proportions were conducted to assess gender differences (girls vs. boys) within each threshold and year. Z-tests were performed using Microsoft Excel (Microsoft Corporation, Redmond, WA, USA) considering significant Z-scores greater than 1.96 or less than − 1.96 and a *p*-value < 0.05. Due to limited sample sizes per country, intra-EU country comparisons were not performed.

## Results

Table 1 shows the levels of EU adolescents engaged in each PIA category for the three years for the whole sample and for boys and girls separately. It also displays the comparative analysis between boys and girls for each PIA category and year. In all cases the percentage of adolescents decreased as the threshold for PIA became more stringent showing a consistent pattern. No gender differences (*p* > 0.05) were found for the “totally inactive” threshold except for the year 2013 (*p* = 0.021; Z-Score = 2.302). On the contrary, a higher percentage of girls than boys (*p* < 0.05) were found in the other three PIA thresholds regardless the analysed year.

**Table 1:** Physical inactivity patterns of European union adolescents among three year-points (2013, 2017 and 2022) for the whole sample as well as boys and girls separately

PIA threshold	Sample	2013		2017		2022	
		*n*	% (CI)	*n*	% (CI)	*n*	% (CI)
Totally inactive	**Whole sample**	570	15.1% (12.3–18.4%)	468	18.6% (15.0–22.0%)	532	14.3% (11.5–17.5%)
	**Boys**	303	13.2% (9.3–17.6%)	251	14.7% (10.3–19.5%	292	12.0% (8.3–15.6%)
	**Girls**	267	17.2% (12.8–21.9%)	217	23.0% (17.4–29.6%)	238	17.2% (12.5–22.5%)
	**Boys vs girls Z-Score**	1.340		2.302		1.712	
	**Boys vs girls ** ***p*** **-value**	0.180		0.021*		0.087	
Very inactive	**Whole sample**	570	39.5% (35.4–43.5%)	468	45.1% (40.6–49.6%)	532	28.0% (24.3–31.6%)
	**Boys**	303	34.3% (28.9–39.7%)	251	37.5% (31.5–43.7%)	292	22.6% (17.9–27.5%)
	**Girls**	267	45.3% (39.9–51.0%)	217	53.9% (47.4–60.8%)	238	34.9% (29.2–41.4%)
	**Boys vs. girls Z-Score**	2.680		3.570		3.126	
	**Boys vs. girls ** ***p*** **-value**	0.007*		< 0.001*		0.002*	
Quite inactive	**Whole sample**	570	58.8% (54.4–63.0%)	468	60.7% (56.2–65.2%)	532	47.7% (43.6–51.9%)
	**Boys**	303	53.8% (47.8–59.4)	251	52.2% (45.8–58.2%)	292	43.5% (37.5–49.5%)
	**Girls**	267	64.4% (58.4–70.5%)	217	70.5% (64.3–76.6%)	238	53.4% (47.6–60.0%)
	**Boys vs. girls Z-Score**	2.571		4.045		2.262	
	**Boys vs. girls ** ***p*** **-value**	0.010*		< 0.001*		0.024*	
Inactive	**Whole sample**	570	74.4% (70.5–77.9%)	468	76.1% (72.0–79.9%)	532	66.5% (62.4–70.3%)
	**Boys**	303	69.3% (64.3–74.3%)	251	69.7% (64.4–75.2%)	292	62.0% (56.4–67.3%)
	**Girls**	267	80.1% (75.1–85.0%)	217	83.4% (78.2–88.4%)	238	72.3% (66.8–78.2%)
	**Boys vs. girls Z-Score**	2.959		3.461		2.497	
	**Boys vs. girls ** ***p*** **-value**	0.003*		0.001*		0.013*	

2013: Eurobarometer 412; 2017: Eurobarometer 472; 2022: Eurobarometer 425

“Totally inactive”: not meeting the minimum of 60 minutes of moderate-to-vigorous physical activity on any day of the week (0 days); “Very inactive”: not meeting the minimum of 60 minutes of moderate-to-vigorous physical activity on at least 3 days per week (<3 days); “Quite inactive”: not meeting the minimum of 60 minutes of moderate-to-vigorous physical activity on at least 5 days per week (<5 days); “Inactive”: not meeting the minimum of 60 minutes of moderate-to-vigorous physical activity on at least 7 days per week (<7 days)

*CI* Confidence Interval

*Significant differences between boys and girls in the Z-Score for two proportions analysis (*p* < 0.05)

Table 2 displays the chi square and standard residuals analysis used to compare the PIA patterns of EU adolescents in 2013, 2017 and 2022. The chi square analysis did not report significant changes (*p* > 0.05) among years in the percentage of adolescents meeting the threshold of “totally inactive” neither for the whole sample nor boys and girls separately. On the contrary, the square analysis reported significant differences among years for the other three PIA threshold (*p* < 0.05) for the whole sample and for girls. In the case of boys this analysis reported significant differences (*p* < 0.05) either for the “very inactive” thresholds or the “quite inactive” threshold, but not (*p* > 0.05) for the “inactive” threshold. On the other hand, for the year 2017 residuals analysis reported a significant increase in the percentage adolescents (whole sample) and girls meeting the “Very inactive” threshold (whole sample [Z-Score = + 4.2]; girls [Z-Score = + 3.4]), the “Quite inactive” threshold (whole sample [Z-Score = + 2.6]; girls [Z-Score = + 2.9]) and the “Inactive” threshold (whole sample [Z-Score = + 2.2]; girls [Z-Score = + 2.1]). In the case of boys this increase was only reported for the “very inactive” threshold (Z-Score = + 2.5). On the contrary, for 2022 the adjusted residuals analysis revealed a significant decrease of the percentage of adolescents (whole sample) and girls meeting the “Very inactive” threshold (whole sample [Z-Score = −5.4]; girls [Z-Score = −3.6]), the “Quite inactive” threshold (whole sample [Z-Score = −4.5]; girls [Z-Score = −3.6]) and the “Inactive” threshold (whole sample [Z-Score = −3.6]; girls [Z-Score = −2.9]). In the case of boys, a significant decrease was observed for the thresholds “very inactive” (Z-Score = −3.9) and “quite inactive” Z-Score = −2.6). Finally, despite the adjusted residuals also suggest a decrease in the percentage of boys in the “inactive” threshold (Z-Score = −2.2), the chi square analysis was not significant (*p* = 0.087).

**Table 2:** Chi square (χ²) and adjusted residuals analysis comparing the physical inactivity patterns of European union adolescents among three year-points (2013, 2017 and 2022) for the whole sample as well as boys and girls separately

PIA threshold	Sample	Adjusted residuals						χ² comparison 2013, 2017 and 2022	
		2013		2017		2022			
		Z-Score	Observed (expected)	Z-Score	Observed (expected)	Z-Score	Observed (expected)	*p*-value	Contingency coefficient
Totally inactive	**Whole sample**	−0.6	86 (93.4)	1.9	87 (74.2)	−1.2	76 (84.4)	0.145	0.049
	**Boys**	0.0	40 (40.1)	0.8	37 (33.2)	−0.8	35 (38.7)	0.640	0.032
	**Girls**	−0.9	46 (50.7)	1.8	50 (41.2)	−0.8	41 (45.2)	0.189	0.189
Very inactive	**Whole sample**	1.4	225 (212.4)	4.2	211 (174.4)	−5.4	149 (198.2)	< 0.001*	0.143
	**Boys**	1.5	104 (94.6)	2.5	94 (78.3)	−3.9	66 (91.1)	< 0.001*	0.136
	**Girls**	0.4	121 (118.7)	3.4	117 (96.5)	−3.6	83 (105.8)	< 0.001*	0.151
Quite inactive	**Whole sample**	1.9	335 (316.9)	2.6	284 (260.2)	−4.5	254 (295.8)	< 0.001*	0.114
	**Boys**	1.8	163 (150.8)	0.9	131 (124.9)	−2.6	127 (145.3)	0.028*	0.092
	**Girls**	0.8	172 (167.2)	2.9	153 (135.9)	−3.6	127 (149.0)	< 0.001*	0.142
Inactive	**Whole sample**	1.4	424 (411.7)	2.2	356 (338.0)	−3.6	354 (384.3)	0.001*	0.092
	**Boys**	1.1	210 (202.7)	1.1	175 (167.9)	−2.2	181 (195.4)	0.087	0.076
	**Girls**	0.8	214 (209.7)	2.1	181 (170.4)	−2.9	172 (186.9)	0.011*	0.111

2013: Eurobarometer 412; 2017: Eurobarometer 472; 2022: Eurobarometer 425

“Totally inactive”: not meeting the minimum of 60 minutes of moderate-to-vigorous physical activity on any day of the week (0 days); “Very inactive”: not meeting the minimum of 60 minutes of moderate-to-vigorous physical activity on at least 3 days per week (<3 days); “Quite inactive”: not meeting the minimum of 60 minutes of moderate-to-vigorous physical activity on at least 5 days per week (<5 days); “Inactive”: not meeting the minimum of 60 minutes of moderate-to-vigorous physical activity on at least 7 days per week (<7 days)

*χ² *Chi Square

*significant differences among years for a given physical inactivity in the chi square test (*p* < 0.05)

## Discussion

This is the first study to examine the PIA patterns of adolescents in the EU using the most recent Special Eurobarometer surveys (2013, 2017, and 2022). The main findings were as follows: (1) regardless of the year, the majority of adolescents did not meet international physical activity (PA) guidelines; (2) girls were more inactive than boys across all PIA thresholds, except for the “completely inactive” category; and (3) the proportion of adolescents in each PIA category increased in 2017 and decreased in 2022, except in the “completely inactive” group which did not report significant changes across the years analysed.

Aligned with previous studies [5, 25, 29] we found that most European adolescents are failing to meet the minimum recommended levels of 60 min of MVPA every day, highlighting the persistent difficulty in achieving sufficient MVPA among this population. While our study does not explore the underlying causes of this inactivity, our findings are consistent with evidence showing that adolescents spend a large proportion of their waking hours engaged in sedentary behaviours—often exceeding 9 h per day [30, 31] and reinforce the need to strengthen PA engagement among EU youth. This is particularly relevant given that physical inactivity is a major modifiable risk factor associated with childhood and adolescent overweight/obesity [32], while global data indicate that youth overweight/obesity has risen markedly and—without coordinated action—is projected to continue increasing towards 2030 and 2050 [33]. Several factors may explain the lower MVPA engagement among 15–17‑year‑olds: increasing academic demands during upper secondary education (ages 16–17), a period marked by high study loads and preparation for final examinations [34]; the widespread availability of sedentary leisure options (e.g. smartphones, video games and streaming platforms) as they may divert time and attention away from PA-related activities [11, 12], particularly among older adolescents who have greater autonomy than younger adolescents; and participation in structured sports appears to decline during adolescence, particularly in late adolescence, due to a combination of increasing academic pressure, shifting social priorities (e.g., romantic relationships), and reduced motivation or access to continue in organised sport settings [35, 36]. Strategies such as improving weekly physical education hours, supporting active recess or active classroom breaks may help to enhance PA engagement among those adolescents in the latest stage of secondary education [37, 38], although additional strategies might also be implemented to promote PA among those adolescents who are not studying. Upstream action in early adolescence (11–14 years) is also warranted, as HBSC surveillance already reports low engagement (up to ~ 80% not meeting guidelines) among 11–15‑year‑olds [8] what seems to persist in the latest adolescence (15 to 17 years) according to our study and previous research [6].

Despite these outcomes, this study observed a significant reduction in the proportion of adolescents not meeting the 60-minute MVPA recommendation in 2022, compared to the two previous time-points, may suggest that the efforts led by the WHO Regional Office for Europe, the European Commission, and national governments are working [1, 2]. This outcome is very positive, not only because changes the increase PIA trend reported in 2017 [39], but also, because it shows an increase in the PA prevalence of European adolescents despite the reported decrease PA among adolescents due to the COVID-19 lockdowns [40]. Nevertheless, while the reported improvement is encouraging, the majority of European adolescents still do not meet the recommended levels of physical activity. Therefore, more effective strategies may be still needed to support adolescents in achieving daily MVPA, especially among girls, who continue to report lower levels of daily engagement in MVPA compared to boys [41]. Furthermore, the observed improvement is based on a single time point (2022), following a previous increase in inactivity in 2017. Therefore, it is premature to interpret our findings as a sustained positive trend, as future Eurobarometer data may reveal a reversal or stagnation of progress. This is particularly relevant considering that the adolescents surveyed in 2022 will not be the same individuals surveyed in future surveys. As such, improvements observed in one cohort may not necessarily translate into long-term behavioural change across generations.

On the other hand, public‑health restrictions and school operations still differed across EU countries during the 2022 Eurobarometer fieldwork, and the fact that the previous Eurobarometer was released in 2017 complicates simple pre–post comparisons and may explain why our findings do not report a decrease in PA in 2022 compared to the pre‑Covid period as in previous research [14, 16]. Against this backdrop, a modest rebound in some behaviours—driven by the resumption of organised sport, reopening of facilities and partial restoration of school routines—could plausibly contribute to the 2022 improvement, even if underlying sedentary habits persist, therefore we again suggest interpreting the reported improvement with caution.

Another major contribution of this paper is the use of different PIA thresholds in order to better understand the PIA behaviours of those European adolescents who are categorised as inactive, and how these PIA behaviours have evolved over a decade. This is particularly relevant, as any engagement in regular physical activity, even below the levels recommended by international guidelines, can yield health benefits for adolescents [3, 23]. Therefore, while increasing the proportion of adolescents who meet the 60-minute MVPA recommendation remains a key objective for EU countries, it is also important to encourage engagement in MVPA on several days of the week [42]. In this regard, our findings show that approximately 15% of European adolescents are unable to engage in 60 min of MVPA on even a single day per week, with no significant changes observed between 2013 and 2022, and no differences between girls and boys. This suggests the existence of a persistent subgroup, regardless of gender, that remains very disengaged from MVPA and may not be effectively reached by current national and international policies [1, 2, 5]. Further research is needed to understand the reasons behind this behaviour as well as to discover how to address deep-rooted barriers that are preventing any engagement in PA among a substantial subgroup of adolescents. This is especially important given the negative impact that no or very low levels of PA throughout the week can have on adolescents’ health [43], and the fact that the PIA behaviour of adolescents is likely to be replicated into adulthood, when the long-term health impacts of physical inactivity become more likely to emerge [44]. Accordingly, these findings underscore the urgent need to develop differentiated strategies that not only increase PA among inactive individuals but also among those adolescents who are not achieving 60 min of MVPA on any day of the week.

Despite no significant changes in the “totally inactive” category between 2013 and 2022, this study reports a significant decrease in the proportion of adolescents classified as “very inactive” and “moderately inactive,” even after the temporary increase observed in 2017. These findings align with the overall reduction in inactivity discussed earlier and may indicate that improvements in adolescent physical activity extend beyond those who were already close to meeting the recommended levels, potentially reaching adolescents with consistently low activity levels. While our analysis is EU‑wide and not designed for country‑level inference, complementary surveillance (e.g. HBSC) consistently reports between‑country gaps in adolescent PA [8]; future research should examine whether the inactivity gradients identified here also display substantial heterogeneity by country, and how that heterogeneity relates to differences in school and community provision. Furthermore, adopting indicators that distinguish subgroups of adolescents according to their weekly MVPA engagement would create policy‑responsive metrics aligned with Global Action Plan on Physical Activity approach and with WHO guidance that any increase below the 60‑minute‑per‑day threshold confers benefit [3, 6]. These indicators could also be embedded in the Eurobarometer and the WHO/Europe Physical Activity Factsheets [45] to enable incremental, country‑comparable targets, with a particular focus on those facing the greatest barriers to accumulating MVPA regularly across the week. In fact, reducing the number of adolescents who achieve fewer than three days per week of 60‑minute MVPA—particularly those reporting zero days—while not sufficient to lower the high prevalence of insufficient activity at the population level, could nonetheless help attenuate health risks associated with physical inactivity and sedentary behaviour in youth [3, 23]. Such incremental shifts are also relevant from a health‑economics perspective, given global projections that physical inactivity will add around US$27 billion per year in treatment costs through 2030 [46]. Moreover, although adolescents who accumulate ≥ 3–6 days/week of 60‑minute MVPA would still be classified as not meeting the youth guideline (≥ 60 min every day), they already reach ≥ 180–360 min/week of MVPA; if this volume is sustained into adulthood, it meets or exceeds the adult recommendation of ≥ 150 min/week— [3, 46]. A life stage in which the burden of noncommunicable diseases is substantially higher than in adolescence [47, 48].

Finally, aligned with previous studies [49] we also found a gender gap among inactive European adolescents, with girls consistently reporting higher levels of PIA across all thresholds and time-points except for the “completely inactive” category. Therefore, further efforts are still needed to enhance the PA engagement among girls and effectively address barriers and factors such as fewer accessible opportunities for girls, gender stereotypes, lower perceived physical competence, and limited social or familial support for engaging in PA [27, 41, 50]. Given the heterogeneity across EU Member State education systems—and recognising that not all adolescents are in school—we frame these as settings‑based actions spanning schools, clubs and community spaces. For those in education, adopting inclusive pedagogy, safe and competence‑building environments, and broadened activity choices, in line with UNESCO’s Quality Physical Education (QPE) guidance, may help to reduce gendered barriers and sustain MVPA among girls [51]. Beyond school, leisure‑time social marketing that addresses fear of judgement and social norms—such as the United Kingdom’s This Girl Can—can complement school‑based efforts by empowering girls and women and helping to normalise physical activity across the life course [52]. In fact, evaluations of this campaign model reported high reach and reductions in perceived judgement among women, although population‑level changes in activity appear context‑dependent [53, 54].

## Conclusions

This study reveals that most European adolescents do not meet minimum MVPA recommendations, with the proportion of “completely inactive” individuals remaining stable at around 15% between 2013 and 2022. While an increase in inactivity was observed in 2017, a significant decline was noted in 2022. However, substantial gender disparities persist, with girls showing higher inactivity levels across all thresholds except for the “completely inactive” category. These findings emphasize the need for more tailored and segmented interventions that consider both baseline activity levels and gender. In this regard, integrating technology-based solutions—such as mobile apps, wearable devices, and interactive digital platforms—may offer effective tools to promote regular PA in adolescents, particularly when embedded within educational and social environments that support sustained and personalized engagement.

### Limitations and strengths

This study has several limitations that must be acknowledged. First, the relatively small adolescent subsample in each survey year limits generalisability and precludes robust trend analyses within individual EU countries; results should therefore be interpreted with caution. Second, the use of different cohorts at each time point may affect comparability across waves; in addition, the inactivity thresholds are not mutually exclusive, which may introduce some classification overlap. Third, EU adolescents’ MVPA was estimated using self‑reported moderate and vigorous activity from the modified IPAQ‑SF. Although the IPAQ‑SF has been validated for populations aged 15–64 years, its accuracy for quantifying MVPA is limited [55]. Moreover, the questionnaire does not capture the full spectrum of youth movement behaviours (e.g. physical education, active play, organised sport, structured exercise, active transport) [29, 40].

Despite these limitations, it is also important to highlight some strengths. First, the dataset derives from official European Commission sources and was collected using a multi‑stage, stratified probability sampling design covering demographic and geographical diversity, which supports external validity. Second, the same questionnaire module and operational rules were applied across the three surveys, supporting comparability over time [39]. Third, the gradient‑based approach to inactivity thresholds provides additional insight into how physical activity patterns may be evolving among European adolescents and underscores the importance of continued monitoring and targeted interventions.

## Data Availability

The datasets reported used and/or analysed during the current study are available from the corresponding author on reasonable request.

## Abbreviations

EU: European Union
PIA: Physically Inactive
MVPA: Moderate-to- Vigorous Physical Activity
PA: Physical Activity
IPAQ-SF: International Physical Activity Questionnaire
WHO: World Health Organization
CI: Confidence Interval
